# Synthesis, microstructural analysis, and wear optimization of Al6061–Si_3_N_4_ composites via stir casting for automotive and aerospace applications

**DOI:** 10.1038/s41598-026-39120-3

**Published:** 2026-02-18

**Authors:** Venugopal M M, Ranjitha P, Vishwanath Koti, Aju Jo Sankarathil, Rajanish M, Kanneganti Jyothishya Brahma Chari, Dayanand M. Goudar, Raju Kandavalli, Hemanth Raju T, Subraya Krishna Bhat, Udayashankar S

**Affiliations:** 1 Department of Aeronautical Engineering , Nitte Meenakshi Institute of Technology, Nitte Deemed to be University, Bangalore, 560064 India; 2 Department of Mechanical Engineering , Dayananda Sagar College of Engineering, Bangalore, 560111 Karnataka India; 3 Department of Mechanical Engineering , Ramaiah Institute of Technology, Bangalore, 560054 Karnataka India; 4 Department of Mechanical Engineering, Saintgits College of Engineering, Kottayam, 686532 Kerala India; 5 Department of Mechanical Engineering , Dayananda Sagar Academy of Technology and Management, Bangalore, 560082 Karnataka India; 6 Department of Civil Engineering, Koneru Lakshmaiah Education Foundation, Vaddeswaram, Andhra Pradesh 522302 India; 7 Department of Mechanical Engineering, Tontadarya College of Engineering, Gadag, 582101 India; 8 Department of Mechanical Engineering, St. Joseph Engineering College, Mangaluru, 575028 India; 9 Department of Mechanical Engineering, New Horizon College of Engineering, Bangalore, 560103 Karnataka India; 10 Manipal Institute of Technology, Manipal Academy of Higher Education, Manipal, India; 11 Department of Mechanical Engineering, VTU, Belgaum, 590018 Karnataka India

**Keywords:** Al6061–Si_3_N_4_ composite, Stir casting, Liquid metallurgy, Wear optimization, Lightweight alloys, Tribological properties, Engineering, Materials science

## Abstract

This study focuses on the synthesis and characterization of Al6061–Silicon Nitride (Si_2_N_4_) composites fabricated using the stir casting technique. The primary objectives were to analyze the microstructure, investigate wear behavior, and optimize wear parameters through the Taguchi statistical approach. Al6061 was chosen as the matrix material due to its lightweight nature, corrosion resistance, and favorable mechanical properties, while 6 wt.% of high-hardness ceramic Si_2_N_4_ served as the reinforcement. The composite was produced via liquid metallurgy to ensure uniform particle distribution and strong interfacial bonding. Scanning Electron Microscopy confirmed homogeneous dispersion of Si_2_N_4_ particles within the matrix. Wear tests were conducted using a pin-on-disc tribometer under varying conditions of load, sliding distance, and speed. Results revealed that the Al6061–6% Si_2_N_4_ composite exhibited significantly lower wear loss compared to the base alloy. The wear of Al6061 alloy and Al6061-6% Silicon Nitride composite at 10 N load, 450 rpm speed and 300 m sliding distance are 91 microns and 115 microns respectively. There is a decrease in wear that is equal to 21% from Al6061 alloy to Al6061-6%Si_3_N_4_ composite. Taguchi L_27_ orthogonal array was used to optimize the process parameters, which showed that Load is the most significant contributing factor i.e., 63.21% followed by Speed 30.38% and Sliding Distance 1.20%. The confirmation tests were carried out for the optimal parameters and the outcomes showed that the error falls in an acceptable range and it is equal to 2.54%. The produced composite resulted in the improvement of microstructure and enhanced tribological properties. These results indicate that the Al6061-Si_3_N_4_ composites are potential materials for automotive, aerospace and wear-resistant engineering parts.

## Introduction

Metal Matrix Composites (MMCs) are a class of materials with characteristics that make them attractive for engineering applications when compared to conventional monolithic alloys, such as increased stiffness, reduced wear and enhanced thermal resistance. The use of aluminum as well as its alloys as matrix materials has garnered a lot of attention on account of their low density, excellent castability and high corrosion resistance. Reinforcements made of ceramic materials, such as boron carbide, silicon nitride (Si_3_N_4_), alumina and silicon carbide for example, effectively enhance wear and mechanical properties of aluminum alloys, making them applicable to aerospace, automotive and military applications^1^.

Al6061 alloy has been widely used as a matrix material of MMCs, since it has reasonable strength, good weldability and can be strongly heat-treatable. The precipitation of Mg_2_Si particles is the main hardening mechanism of Al6061. This behavior can be improved through the introduction of strong ceramic reinforcements^2^. However, achieving a homogeneous dispersion and strong matrix-reinforcement interface is still a major challenge. Silicon nitride, also referred to as Si_3_N_4_, is a type of ceramic that does not include any oxides and is commonly recognized for its remarkable hardness, good elastic modulus and outstanding wear resistance. Its low density (~ 3.2 g/cm^3^) and high thermal stability make it an attractive reinforcement for lightweight aluminum alloys^3^. The introduction of hard particulate reinforcement such as Si_2_N_4_ was reported to cause grain refinement by Zener pinning effect, resulting in the suppression of grain growth during solidification and heat treatment, which helps improve yield strength via Hall–Petch phenomenon. Likewise, reinforcement particles impose localized stresses to interact with dislocations, resulting in a higher density of dislocations and a decrease in their mobility to provide a dislocation strengthening and superior plastic deformation resistance.

The importance of quantitatively characterizing these microstructures features has been emphasized by recent aluminum composite studies. Through advanced microscopy investigation, it was observed that Si_2_N_4_ reinforcements can lead to the more homogeneous microstructure with finer grains and stronger bonding interfaces of which can eliminate a localized stress concentration and improve the load transfer across the matrix-particle interface ^4^. The latter are different than composites with traditional reinforcements like SiC which, in general, suffer from agglomeration/weak interfaces effects that compromise strengthening efficiency. Therefore, in order to link these microstructural states with the observed wear mechanisms and mechanical response, quantifiable measures of particle dispersion, grain boundary character and local defect structures (i.e., low- and high-angle boundaries; subgrain networks) are necessary.

The processing pathway for metal matrix composites (MMCs) greatly affects the microstructural evolution, reinforcement distribution, interfacial bonding, defect formation and therefore mechanical and tribological performance. Among liquid-state processes, stir casting is still one of the most used fabrication methods for aluminum composites and can be considered an easy, cheap and scalable method for large components ^5^. Stir casting enables dispersion of discontinuous ceramic particulates (e.g., SiC, Al₂O_2_, Si_2_N_4_) into molten aluminum such that composites can be generated with relatively homogenous reinforcement distribution and better property coupling versus non-reinforced alloys if the process parameters are controlled. Nonetheless, problems such as agglomerations of particles, porosity formation and an discontinuity of the interfacial wetting remains, one is generally required to carefully control conditions, such as rotation speed of a stirrer, temperatures for melting process and a design configuration or pattern of an impeller and a method of adding reinforcement powder that would need to be added with other processing variables in order to obtain homogeneous-performance products^6^. Recent investigations are still attempting to understand these processing variables and their effects on microstructure and performance in a variety of Al-alloy MMCs manufactured using stir casting, or similar techniques. For instance, optimization at the stirring casting variables using the response surface methodology and design of experiments (DOE), was demonstrated to have some influence on mechanical properties of TiO_2_-Al7075 composites, highlighting dataset sensitivity to microstructure as a result of casting.

Apart from the conventional stir casting, advanced synthesis systems are under serious consideration as an alternative to address limitations associated with melt based methods. Advanced solid-state techniques such as FSP and friction stir additive manufacturing have found application for aluminum based MMCs, wherein owing to severe plastic deformation and dynamic recrystallization caused by the tool, grain refinement is facilitated; Improvement in reinforcement dispersion and elimination of defects is achieved without melting the matrix^7^. The fact, for instance, that multi-pass FSP has been shown to homogenize microstructures and significantly enhance wear resistance and mechanical properties in SiC-reinforced aluminum composites highlights the role of such processing strategy on structure–property relationships. Moreover, hybrid and hybrid-assisted fabrication pathways-e.g., stir casting followed by ultrasonic stirring, squeeze casting or friction stir post-processing are being pursued to reduce particle clustering and increase interfacial bonding; with recent works that reported better microstructure control as well as improved corrosion performance of aluminum nanocomposites prepared via ultrasonically-assisted squeeze casting^8^. These emergent processing approaches represent a larger trend in the field of MMCs toward uniting process design, microstructure engineering, tribological performance and statistical optimization to create a more holistic decision-making framework for MMC development than what could be accomplished through trial-and-error casting alone. This would provide important insight into how processing effects wear behavior and microstructural stability in modem materials research, such as Al6061-Si_2_N_4_ composites.

Wear behavior is an essential property to evaluate the potential application of MMCs in tribological conditions. The wear performance of the developed composites was evaluated under different load, speed and sliding distance conditions on a pin-on-disc tribometer^9^. The study aims to discuss the influence on wear resistance and frictional behavior of Si_2_N_4_ reinforcement in comparison with the base Al6061 alloy.

When the wear procedure is carried out, some characteristic parameters have to interact with each other, like the applied load, sliding speed and sliding distance. To analyze and optimize these variables in a systematic way, the Taguchi DOE technique was employed^10^. This statistical approach uses an orthogonal array in order to minimize the number of tests needed, while maximizing possibilities for information. Both the signal-to-noise ratio (S/N) and analysis of variance (ANOVA) were used to determine the influential factors that affected the wear. The optimization results provide a scientific basis for the attainment of maximum wear performance for composites comprising Al6061-Si_2_N_4_. The pure data-driven ML models generally need large and high-quality datasets as well as careful feature selection to prevent over fitting and maintain physical interpretability, which is difficult in tribology due to limited experimental tribology data and expensive fully developed wear tests^11^. On the other hand, the Taguchi method is still a useful and solid methodology for experimental design and screening of initial parameters, especially when the number of factors is low to moderate and main effects analysis is pursued. Taguchi design effectively minimizes the number of experimental runs, maintains orthogonality among factors and yields easy to interpret signal-to-noise analysis which is useful for determining dominant wear parameters in MMCs^12^. Here, Taguchi is positioned as an adjunctive screening and optimization technique that can be paired with RSM or follow-up ML regression models to reduce predictive error and create hybrid modeling approaches that are statistically sound yet have some mechanical basis.

Metal matrix composites (MMCs) based on Al6061 alloy reinforced with silicon nitride (Si_3_ N_4_) ceramic particulates have been extensively studied because they combine the high strength to low density ratio, good corrosion resistance, and superior mechanical and tribological properties. Si_2_N_4_ reinforcement, as observed in our past study, improved the hardness, wear resistance and load carrying capacity of Al6061 composites produced via stir casting-based processing routes^13^. However, and despite this extensive literature, the majority of these studies have focused on establishing general trends in wear behavior or mechanical performance but have not systematically optimized the wear parameters for a range of reinforcement contents.

Especially, the combined effect of important tribological parameters like applied load, sliding speeds, and sliding distance on wear property of Al6061-Si_2_N_4_ composites at certain degrees of reinforcement weight percentage has not been systematically optimized in robust statistical design protocols^14^. Furthermore, most of the contemporary research focuses on the macroscopic wear loss data and they explain the wear-induced surface mechanisms.

Thus, the objective of this study is to fulfill the existing gap by systematic optimization of wear parameters for Al6061-Si_2_N_4_ MMCs with selected Si_2_N_4_ weight fractions using Taguchi technique. This is supplemented with extensive microstructural and worn-surface analysis to develop well-defined structure–property-wear relationships. The study does not only claim novelty in material selection, but is mainly focused on a process relevant interplay between statistical optimization and microstructural validation.

The literature review on synthesis, microstructural characterization and wear analysis of particle reinforced composites of Al6061 is presented below.

Nagesh et al.^15^ examined the sliding wear characteristics of Al-B-Gr composites. It was noticed that Al6061-B-Gr composites were successfully fabricated through stir casting. From the experimental findings, it was found that both the load and sliding speed of specimen have obvious influence on wear in sliding. Muthu^16^ et al. analyzed the wear performance of Al6061 Alloy-TiC-Basalt MMCs. From ANOVA, it is observed that sliding distance followed by load and sliding velocity are the first three dominant factors. The wear performance of Al6061-SiC composites were studied by Gonfa et al.^17^ and multi-objective optimization was done. The S/N ratio analysis showed that the inclusion of SiC in the composite material increased its strength and wear resistance.

Bhave et al.^18^ performed fabrication and wear test of AA6061-B_4_C composites. From the ANOVA results, it was revealed that the load and sliding distance were both statistically significant factors affecting wear volume. Iyengar et al.^19^ studied the wear property composite Al6061-TiB_2_-CeO_2_ under Taguchi approach. From these results it was found that the addition of TiB_2_ had significant influence over the wear of the composites. Padmaraj et al.^20^ investigated the wear behavior of Al6061-B_4_C-Novel Squid Quill Ash Composites. Statistical investigation revealed that load is a major parameter on the wear performance of the composites. This is along with the percentage of SQA and sliding speed, which were also investigated.

Rao and Nallu^21^ worked on optimizing the wear parameters for Al6061 alloy-TiB_2_ MMCs. It was found that the findings of the confirmation experiments conducted fitted within 10% of the predicted value. The ANOVA results indicated that speed was the most significant processing parameter to be optimized. Singh and Gupta^22^ applied Response Surface Methodology to obtain information about the wear behavior of Al6061 MMCs. The sliding velocity was determined to be the dominant factor governing the wear rate of synthesized composites, as revealed by an ANOVA. Kumar and Reddy^23^ examined the wear performance of Al6061-Si_3_N_4_-SSP-RHA composite. From the ANOVA findings, it was found that the major parameter when predicting a particular wear rate is speed, then load and reinforcement. Sama et al.^24^ investigated the wear properties of Al6082 + BN + B_4_C + Corn Cob Ash composite. According to the outcomes of the ANOVA, the parameter that has the most significant impact is the load, which is responsible for 51.6% of the total occurrence.

The SiC-Si_3_N_4_-BN-Al_2_O_3_ composite was found to have excellent mechanical properties, such as high hardness and abrasion resistance and low coefficient of friction^25^. From the results, it was observed that the desired prototype alloys superior mechanical properties have been experimentally produced, and an excellent match was achieved between predicted optimal parameter settings and final properties^26^.

The findings show promising response, indicating relatively decreased wear and a higher value of frictional coefficient under dry sliding. This unusual system of the developed material could be a potential candidate for alternative brake and clutch materials systems. With the proposed model, the wear evolution process of modified gear rack under mixed elasto-hydrodynamic lubrication (EHL) is adequately predicted by a cyclic iterative method to obtain the real-time contact features with wear degradation of tooth profile^27^. Systematic ball-on-disk tribological measurements demonstrated a good tending between tribological performance and output currents, the M-TC showed an ultralow friction coefficient of 0.066 in self-lubricating state and a remarkable peak power density of 1.18 kW m^−2^.

A number of research on Al6061-SiC composites developed by stir casting have revealed wear reductions which are generally in the range of 20–40% according to intensity content, size and condition used for testing. Although the hardness improvement by SiC is substantial, its angular shape would most of the time increase abrasive action with the counterface at elevated loads and may cause unstable wear^28,29^. Nevertheless, in practice the application of such systems often faces issues with poor wetting behavior, higher processing temperatures and more expensive materials.

For the Al6061-TiB₂ composites, the reported wear enhancements range from 25 to 45%. TiB₂ has good interfacial bonding and thermal stability, but in-situ synthesis methods are frequently necessary to achieve uniform dispersion leading to more complex processing^30^. In this context, the recorded 26.37% wear decrease of Al6061-Si_2_N_4_ composites is competitive with those presented in TiB₂-based systems as well as similar to that obtained in low-end SiC-reinforced composites and produced through a relatively easy stir casting procedure. Although inferior to that observed in some B_4_C-reinforced systems, the use of Si_2_N_4_ has been shown to have benefits in terms of chemical stability with aluminum, lack of formation of detrimental reaction phases, and retention of tribolayer under moderate loads.

Al6061 based MMCs have been widely studied because of their excellent strength-to-weight-ratio, corrosion resistance and industrial applications. Initial studies were mainly dedicated to reinforcing Al6061 with the traditional ceramic particles, like SiC, Al₂O_2_, B_4_C and TiB₂ in order to improve mechanical and tribological properties^31^. These studies identified the importance of hard second phase particles for hardness and wear resistance enhancement because of load transfer and abrasion-based mechanisms. However, most of these studies featured simplified parametric investigations that concluded an increase in wear resistance with monotonically improving microstructure effects without consideration for microstructural-wear interactions or stability over different service conditions.

Recent trends in tribological research emphasize wear mechanism transitions, formation of the tribolayer and non-linear wear behavior for varying load-sliding conditions. Subsequent research has proven that wear of MMCs is controlled not only by the amount of reinforcement, but also depends on the interplay between microstructural stability, debris formation and contact stress. Nevertheless, Al6061 system using Si_2_N_4_ as a reinforcement phase has received much less attention and even fewer are related to systematical wear optimization^32,33^. Si_3_N_4_ has several advantages compared to the traditional reinforcements because of its high thermal stability, chemical inertness, low density and smaller interfacial reaction with aluminum. These features indicate their potential for the enhancement of stable tribo-film formation and to reduce severe adhesive wear, however minimal attention has been given to wear modelling with a mechanism-based explanation, as existing studies are mainly concentrated on mechanical properties.

From a process standpoint, stirred casting is still the most commercially viable method for producing aluminum MMCs. Although there is no lack of literature describing stir casting as a general process, recent work has shown that even small differences in the processing conditions-melt superheat, particle preheat and stirring conditions in particular-can have a profound effect on the dispersion of reinforcement phases, porosity and interfacial bonding^34^. However, the majority of published work situates processing conditions as qualitative descriptors, rather than quantitatively and systematically relating them to tribological behaviors thus a hole has persisted in generating strong structure–property-performance relationships.

Statistical optimization methods, such as the Taguchi method, have been used for identifying the main wear parameters in tribological research. Nevertheless, many of the existing literature uses Taguchi designs without proper experimental verification and replication, as well as without an interpretation in terms of mechanism; they frequently report high signal-to-noise ratios that are not linked to wear mechanisms or microstructural features. In addition, the recent progress in regression- and machine-learning-based wear prediction indicates that the requirement of physically interpretable model is not negligible especially if only few experimental data are available^35,36^. In this sense, the Taguchi technique of parameter optimization is still an acceptable and convenient method for wear screen testing when it is supplemented by extensive data on wear, microstructure evidence and repeat experiments.

The above critical review of the literature indicates a clear void in the combined investigation of stir cast Al6061-Si_2_N_4_ composites involving organized wear testing, statistically attested optimization and mechanism based microstructural analysis. The present study attempts to bridge this gap by exploring the optimal tribological parameters at a constant practical reinforcement level, while explicitly linking wear behavior with microstructural features and transitions in wear mechanisms. This work goes beyond the repeated material property relation reporting and adds extra insights on wear performance in Si_2_N_4_-reinforced aluminum MMCs. The developed composites are widely used in applications such as automobile, aeronautics, marine to industrial and electronic.

## Materials and experimental details

### Materials

Commercial Al6061 aluminum alloy was employed as the matrix. Al6061 matrix was procured from Fenfi Metallurgicals Bengaluru, Karnataka. Among the precipitation-hardening aluminum (Al) alloys, Al6061 is a member of the Al–Mg-Si series and possesses a moderate level of strength. Due to its superior strength, weldability and machinability, it is one of the most extensively applied aluminum alloy products^37^. Strength is mainly derived from Mg₂Si precipitates that are formed during artificial aging. Good strength-to-weight ratio making it suitable for structural and automotive applications. The constituents of the Al6061 alloy are reported in Table 1.

**Table 1:** Elements of Al6061 Alloy.

Constituent	Amount in percentage
Al	Balance
Si	0.6
Zn	0.14
Mn	0.07
Cr	0.15
Ti	0.09
Fe	0.4
Cu	0.25
Mg	0.9
Others	0.02

The reinforcing material employed in the above investigation was silicon nitride, also referred to as Si_3_N_4_. The Si_2_N_4_ particles were obtained from Vasa Scientific, Bengaluru, Karnataka, India having purity ≥ 99% and particle size of 40 µm to impart thermal stability during the melt processing and enhanced load transfer in aluminum matrix. Before introducing composites, the Si_2_N_4_ powders were preheated to eliminate water and enhance wettability with molten Aluminum alloy. Silicon nitride, a family of ceramics that do not contain any oxides, are well known for their higher hardness, lower density and excellent wear resistance. It is listed as one of the key advanced ceramics used in elevated temperature and higher stress applications^38^. In metal matrix composites (MMCs), the reinforcement of Si_2_N_4_ substantially enhances the mechanical strength, hardness as well as wear resistance of the aluminum matrix. Moreover, this improvement is obtained without an excess of weight and exhibits good thermal shock resistance.

### Experimental details

The Al6061 alloy ingots were melted in an electric resistance furnace at 750 °C under atmospheric conditions, and the molten alloy was degassed with hexachloroethane tablets to reduce porosity. The Si_2_N_4_ reinforcement particles were pre-heated at 500 °C for 30 min to improve wettability and to minimize thermal gradients during addition^39^. The Si_2_N_4_ content of the reinforcement was 6 wt. % (based on previously reported optimum limits) for stir-cast Al6061 composites and capable of exhibiting good wear resistance without creating porosity, particle agglomeration or poor wettability. This choice guaranteed the consistency of grain structure and effective adjustability of tribological parameters. The stirring was performed mechanically with a graphite-coated stainless-steel impeller at 400 rpm for 10 min to achieve small and uniformly dispersed particles. The mixed melt was cast into a preheated metal mold kept at about 300 °C to minimize the solidification shrinkage. The pouring temperature was set at 750 °C and once solidified, the cast samples were taken out of the mold and air-cooled to room temperature. All the samples utilized in the current study were examined as-cast.

**Fig. 1:**
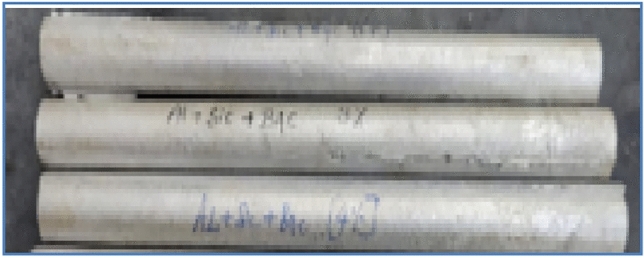
Casted Al6061-6%Si_3_N_4_ composite.

The specimens for microstructural investigation were cut from the casting composites and embedded in epoxy resin. The samples were prepared for standard metallographic procedures with grinding on SiC papers up to 800 and diamond suspensions down to 1 µm finish. The polished specimens were etched by Keller’s reagent to indicate matrix-reinforcement interface^11^. Microstructural observation was conducted with a scanning electron microscope (SEM) at 20 kV. The existence and distribution of Si_2_N_4_ particles in Al6061 matrix were also verified by SEM. The cast Al6061-6%Silicon Nitride composite is shown in Fig. 1.

Dry sliding wear experiments were performed on a pin-on-disc tribometer in compliance with the ASTM G99 standard. Cylindrical pin samples of 8 mm X 30 mm were cut from the cast composites. The counterface disc material was EN31 steel, having hardness of 60 HRC with mean surface roughness of 0.6 µm. Wear tests were conducted under controlled laboratory environmental conditions at room temperature^40^. The test conditions, including the normal load, sliding velocity and stroke length were changed according to the experiment design. The pin and disc surfaces were cleaned with acetone to eliminate possible contaminants prior to each test. Wear in microns was recorded.

The Taguchi approach was used to optimize the wear properties of Al6061–Si_2_N_4_ composites by studying in a systematic manner the effect of applied load, sliding velocity and sliding distance. An L27 OA was utilized, which has 3 control factors at 3 levels each allowing for the study of main effects and interaction trends with limited amount of experiments. The ‘smaller-the-better’ S/N ratio was used, because our main goal in this study was to minimize the wear^41^. This condition is justified for tribological end-conditions where wear loss directly translates to performance gain. All the experimental conditions were repeated three times and the wear averaged to ensure confidence in data. A one-way ANOVA was carried out by using the average S/N ratios to determine significance of the effect of each factor, and the pure error was calculated from repeated runs.

**Fig. 2:**
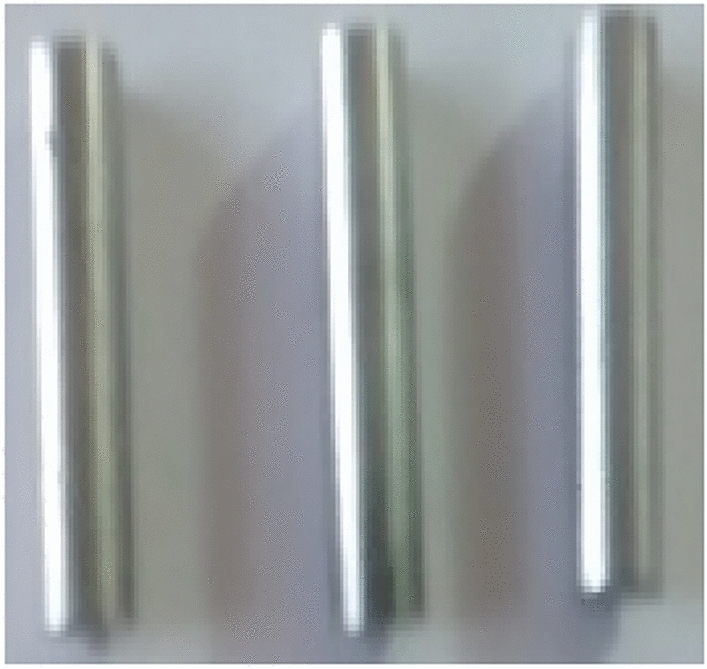
Wear test samples.

The samples for wear testing are illustrated in Fig. 2.

Minitab software was used to find the best values for the process variables that affect how any system works. Table 2 indicates the design variables and their corresponding values employed in the current study. The wear (in microns) of Al6061-Si_3_N_4_ composites was examined as the response variable. Table 3 displays the design of experiments in accordance with L_27_ orthogonal array.

**Table 2:** Design variables and their level.

Design factors		Levels		
		1	2	3
Load	(N)	10	15	20
Speed	(Rpm)	150	300	450
Sliding Distance	(m)	300	600	900

**Table 3:** Design of experiments in accordance with L27 orthogonal array.

Sl. No	Load (L)	Speed (S)	Sliding distance (D)
1	10	150	300
2	10	150	600
3	10	150	900
4	10	300	300
5	10	300	600
6	10	300	900
7	10	450	300
8	10	450	600
9	10	450	900
10	15	150	300
11	15	150	600
12	15	150	900
13	15	300	300
14	15	300	600
15	15	300	900
16	15	450	300
17	15	450	600
18	15	450	900
19	20	150	300
20	20	150	600
21	20	150	900
22	20	300	300
23	20	300	600
24	20	300	900
25	20	450	300
26	20	450	600
27	20	450	900

## Results and siscussion

### XRD study of Al6061 alloy-6% silicon nitride particulate composites

**Fig. 3:**
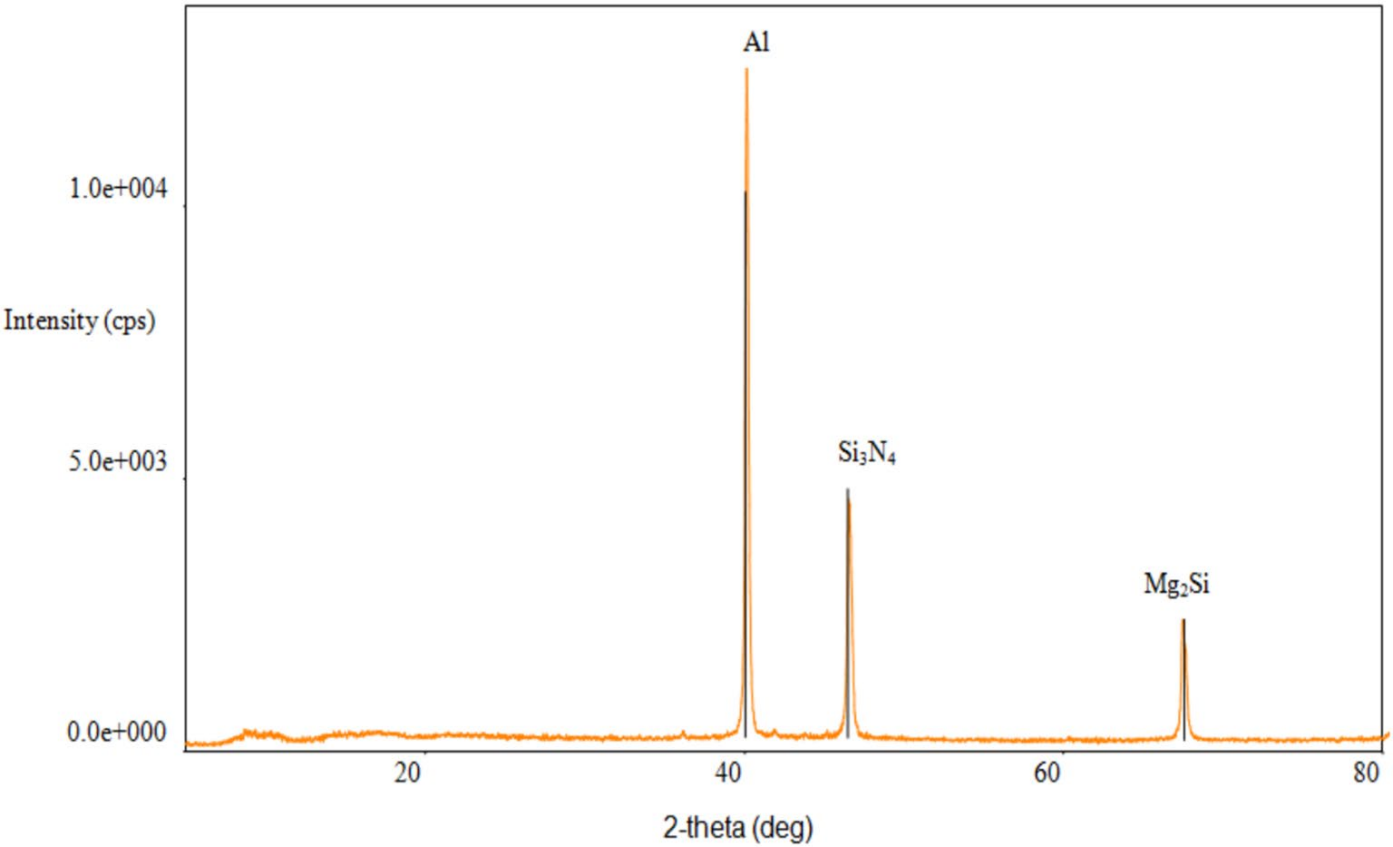
XRD spectrum of Al6061-6%Si_3_N_4_ composites.

The X-ray diffraction analysis of composites made of Al6061-6%Si_3_N_4_ is depicted in Fig. 3. The XRD pattern of Al6061-Si3N4 composite shows α-Al and Mg_2_Si peaks. Further diffraction peaks related to Si_2_N_4_ can be seen. These peaks compare well with the standard JCPDS data of hexagonal β-Si_2_N_4_, verifying that the ceramic reinforcement was successfully embedded and is in good phase during stir casting. The lower Si_3_N_4_ intensity peaks could be due to the lower weight fraction and finer particulates of silicon nitride relative to the aluminum matrix^42^. No further reaction products, such as Al_4_C_3_ or AlN were observed within the resolution of XRD measurement suggesting no harmful interfacial reactions occurred between the Al6061 matrix and Si_2_N_4_ reinforcement during processing. This validates thermodynamic compatibility between Si_2_N_4_ and the aluminum matrix at the chosen casting conditions.

### Microstructure study of Al6061 alloy and Al6061-6%Si_3_N_4_ composites using SEM

**Fig. 4:**
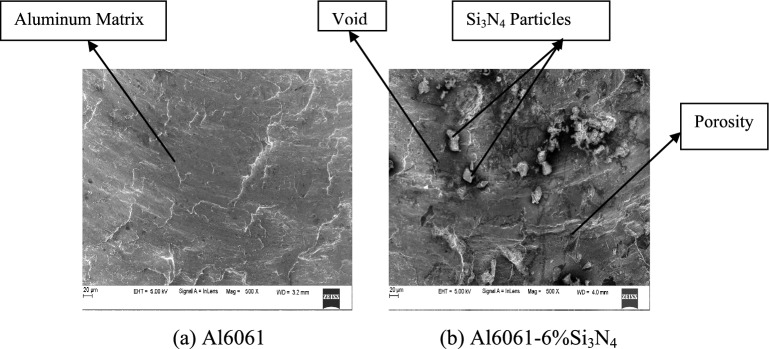
SEM Images of aluminum matrix and Al6061-6%Si_3_N_4_ composite.

The SEM images of Al6061 aluminum alloy and Al6061 alloy-6%Si_3_N_4_ composites are indicated in Figs. 4 (a) and (b) correspondingly. The base alloy microstructure reveals a coarse matrix with regions of plastic flow localization, whereas the composite contains silicon nitride particles embedded in the aluminum matrix^43^. The particles are uniformly dispersed in the matrix, although local clustering of them cannot be entirely ruled out at this magnification.

The SEM micrographs indicate that there is only mechanical interlocking for the matrix-reinforcement interface, and no distinct gaps or interfacial regions with significant particle pull-out observed at the analyzed areas. Although such observations indicate fairly good particle incorporation by the stir casting, no quantitative evaluation of porosity, grain size and distribution was carried out in this investigation. Thus, statements about uniform distribution and grain refinement are qualitative only interpretations of the micrographs.

To support the microstructural evidence, quantitative analysis was performed on selected SEM Micrographs at the same magnification of each image using ImageJ Software. The Si_2_N_4_ particle spatial coordinates in terms of inter-particle distance and particle area fraction were established. The analysis reveals that the Si_2_N_4_ particles are well dispersed in the Al6061 matrix and average area fraction of particles is approximately equal to nominal reinforcement content^44^. Some minor agglomeration at near full reinforcement levels was also identified, but small scale and infrequent, with no microstructure dominance.

On etched SEM images, the grain sizes were measured by the linear intercept method. An obvious grain refinement is presented in the Si_2_N_4_ reinforced composites when compared to the unreinforced Al6061 alloy. The average grain size declined with increasing Si_2_N_4_ content due to the pinning effect of ceramic particles on the grain boundary movement during solidification. Porosity was then analyzed using threshold-based image segmentation in ImageJ. The measured percentage porosity was relatively low in all composites and increased only incrementally with reinforcement content. The low porosity is suggestive of adequate degassing and controlled solidification during stir casting.

### Study of Worn surfaces of Al6061-6%Si_3_N_4_ composites using SEM

**Fig. 5:**
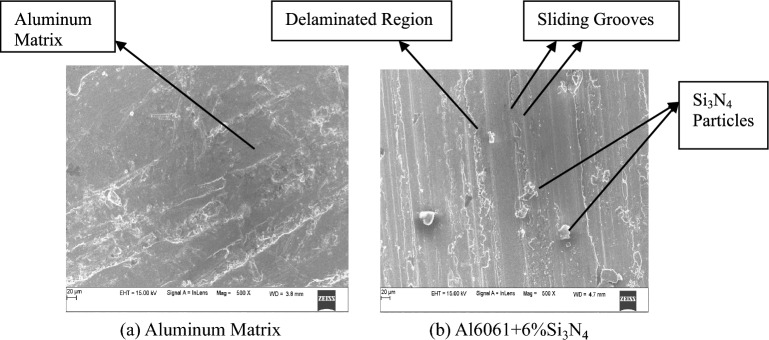
(**a**) Aluminum matrix, (**b**) Al6061 + 6%Si_3_N_4_.

Figure 5 Worn Surfaces SEM Images of Aluminum Matrix and Al6061-6% Si_3_N_4_ composite.

The SEM Photographs of worn surfaces of Al6061 alloy and Al6061-6%Si_3_N_4_ are shown in Figs. 5a and b respectively. The worn surface of plain Al6061 alloy is identified by severe plastic deformation, significant material transfer and deep groove shape parallel to sliding direction. These characteristics are a sign of predominant adhesive wear due to the high propensity of the ductile aluminum matrix to adhere to the steel counterface ^45^. Localized material tearing and smearing indicates repetitive junction formation and breakdown, which correlates with the high wear rate of the base alloy.

The wear morphology for the Al6061-Si_2_N_4_ composites is quite different. The surfaces contain relatively shallower grooves with less plastic flow, and fragmented Si_2_N_4_ particles are embedded in the wear track. Such features would suggest that wear had shifted from being dominated by adhesion to a combination of abrasion and adhesion. The hard Si_2_N_4_ particles are the load-carrying material when direct metallic contact is restricted and severe adhesion is prevented^46^. Localized areas of particle debonding and delamination can be seen in the composites, especially at higher loadings. But these characteristics are local or space-constrained and do not control the whole wear surface. The existence of these local damage sites is attributed to stress concentration at the matrix-particle interface under sliding. Significantly, notwithstanding some isolated debonding phenomenon, the overall wear rate of the composites is kept lower than that for the primitive alloy. It appears that the efficient load-transfer and surface contact are more effective to degrade the local damage mechanisms than detrimental. In addition, the fragmented Si_2_N_4_ particles also favor a tribolayer formation which is helpful in stabilizing the sliding interface and therefore reducing the material loss^47^. Such tribolayer formation accounts for the enhanced wear resistance of the composites despite some delamination features.

### Optimization of wear process variables using Taguchi experimental design technique for Al6061-aluminum oxide composites

A series of 27 experiments were carried out to ascertain the influence of various variables on the wear of Al6061 and Si_3_N_4_ composites. The trials were carried out utilizing the L_27_ orthogonal array, and the wear outcomes are displayed in Table 4. The wear of Al6061 alloy and Al6061-6% Silicon Nitride composite at 10 N load, 450 rpm speed and 300 m sliding distance are 91 microns and 115 microns respectively. There is a decrease in wear that is equal to 21% from Al6061 alloy to Al6061-6%Si_3_N_4_ composite.

**Table 4:** DOE of L_27_ orthogonal array (OA) and wear outcomes.

Sl. No	Load (L)	Speed (S)	Sliding distance (D)	Wear (microns)
1	10	150	300	95
2	10	150	600	89
3	10	150	900	82
4	10	300	300	104
5	10	300	600	98
6	10	300	900	92
7	10	450	300	115
8	10	450	600	118
9	10	450	900	122
10	15	150	300	45
11	15	150	600	42
12	15	150	900	34
13	15	300	300	58
14	15	300	600	52
15	15	300	900	45
16	15	450	300	84
17	15	450	600	87
18	15	450	900	90
19	20	150	300	53
20	20	150	600	49
21	20	150	900	47
22	20	300	300	65
23	20	300	600	59
24	20	300	900	54
25	20	450	300	75
26	20	450	600	71
27	20	450	900	68

The wear findings for Al6061 and Si_3_N_4_ composites are shown in the above table. The ANOVA result for wear is indicated in Table 5. This study was undergone for 5% significance level and 95% confidence level^48^. This indicates that, among the wear test criteria, speed has the most impact on the wear response.

**Table 5:** ANOVA outputs for weight loss.

Source	DOF	Seq. SS	Adj. SS	Adj. MS	F	p	Contribution %	Inference
Load	2	10,473.2	10,473.2	5236.6	121.71	0.000	63.21	Significant
Speed	2	5034.3	5034.3	2517.1	58.50	0.000	30.38	Significant
Sliding distance	2	200.1	200.1	100.0	2.33	0.124	1.20	Insignificant
Error	20	860.5	860.5	43.0			5.193	
Total	26	16,568.1	16,568.1				100	

R-Sq = 94.81%

The R^2^ value of 94.81% in Taguchi analysis of Al6061-Si_2_N_4_ composites indicates that the developed regression or ANOVA model accurately represents the relationship between wear rate and process parameters (load, speed, and distance). This high value validates that your experimental design, data consistency, and parameter optimization are scientifically sound and reliable.

The analysis of ANOVA outcomes for wear in response to the factors are presented in the table above. The outcomes reveal that applied has the major impact, contributing 63.21%, and then followed by Speed, which accounts for 30.38%, and Sliding Distance, which accounts for 1.20%. Thus, this factor shows an excellent improvement in the results^49^.

The low percentage contribution of sliding distance (1.2%) to the total wear response suggests that over the range of experiments carried out, wear in Al6061-Si_2_N_4_ composite system does not just follow the increase in the cumulative sliding length and is predominated more by load-dominated contact mechanics and surface stability. In the tribological systems of reinforced aluminum MMCs, sliding distance has a significant impact on wear development only when the controlling wear mechanisms are formed. In the instant case, after an initial wear regime, the wear proceeds rapidly to a steady state regime during which a mechanically mixed tribolayer comprising compacted debris and fractured Si_2_N_4_ particles is formed^50^. After the formation of the tribolayer, subsequent sliding will not lead to a higher rate of material removal by an analogous mass but by one that is far lower and thus is one which also has significantly weaker dependence on sliding distance.

Applied load exerts dominant control on the real area of contact and normal stress magnitude at the sliding surface, which are the important factors that determine wear behavior among the parameters studied. At lower applied loads, the contact pressure is still not enough to initiate more significant plastic deformations of the Al6061 matrix. With further increment of the applied load to medium levels, contact stress is higher than yield strength of aluminum matrix for promoting local plastic deformation. Under this regime, the hard Si_2_N_4_ receives full play as loading members. This load-accelerated activation of reinforcement effectiveness accounts for the decrease of the wear rate with an increasing load^51,52^. However, at higher applied loads the contact stress exceeds the interfacial strength between matrix and loading fiber, initiating subsurface cracking, particle debonding and delamination. The protective tribolayer caring here breaks down, leading to cycles of breaking the layer and loading new soft matrix face material. Meanwhile, extreme plastic deformation of the matrix exerts material removal effect by abrasive and delamination wear. As a result, the wear rate starts to grow steeply under this high-load condition.

**Fig. 6:**
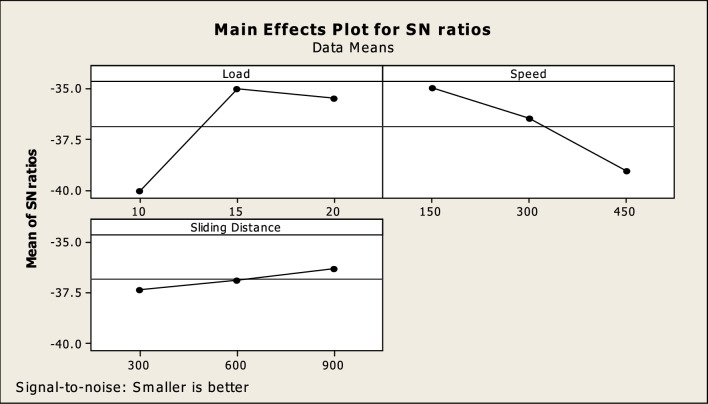
Main effect plot for SN ratios.

Figure 6 shows the major effect diagram for wear after the tests were conducted according to Taguchi’s experimental methodology. Depending on the main effect, the response and its dependent parameters are affected directly.

The above graph has been plotted on the basis of smaller-the-better theory. The above plot indicates that as load increases the wear increases up to level 2 and then decreases slightly at level 3. As speed increases the wear decreases from level 1 to level 3. Additionally, the wear rises from level one to level three as the sliding distance moves further apart^53^. The level one in load, the level three in speed, and the level two in sliding distance were found to be the optimal average response values for wear.

Figures 6, 9 and 10 show the non-monotonic wear behavior; initial decrease in wear with increasing load followed by increase at higher applied loads. This phenomenon is attributed to the switch of the prevailing wear mechanisms and the stability of the compositionally modified surface layer formed during sliding^54^. At lower loads, wear is excess because of inadequate sintering of the worn particles and insufficient formation of protective tribofilm. When the applied load increases to a moderate level, contact pressure helps compact Si_2_N_4_ particulates and oxidized wear-debris in sliding interface. As a result, wear reduces with an increase in the applied load. This regime is attributed to mild abrasive wear with significant load-sharing by the hard Si_2_N_4_ reinforcement.

At higher loads, the contact stress applied exceeds the mechanical strength of tribolayer. The protective film is repeatedly broken and delaminated, resulting in the exposure of fresh matrix material to the counterface. At the same time, higher subsurface stresses induce particle debonding and microcrack nucleation at the matrix-reinforcement interface^55^. These effects lead to an increased material removal rate via intense abrasion and delamination, which in turn increases wear rate. Therefore, the changing trend of wear rate is also suggested to reveal a transition from low-load induced adhesive wear to middle-load controlled mild wear for the worn surface and finally up to heavy abrasive-delamination dominated severe wear.

**Fig. 7:**
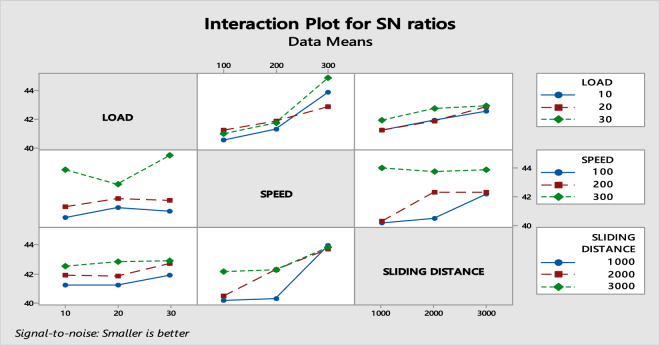
Interaction plot for SN ratios.

Figure 7 indicates the illustration of the interaction impact that components have on wear outcome. It can be seen from the interaction graph that follows that the impact of a single parameter on the wear is greater, while the impact of two parameters interacting with each other has a smaller impact on the wear^56^.

Table 6 indicates the delta value. The delta value reveals the change in mean for the selected levels. The delta value is obtained by taking the difference between the minimum average values from the maximum average value of S/N ratio^57^. The factor having maximum delta value is having major impact on weight loss. The value of delta for the wear highlights the reality that load is the major component in terms of importance.

**Table 6:** Delta values.

Levels	Loads	Speed	Sliding distance
1	− 40.07	− 34.97	− 37.35
2	− 35.02	− 36.49	− 36.90
3	− 35.4	− 39.10	− 36.31
Delta	5.05	4.13	1.04
Rank	1	2	3

A response table for wear response is shown in the above table. Based on the information presented in this table, it can be deduced that the variable that has the greatest impact on the Al6061-Si_3_N_4_ composites is that of Load, followed by Speed and Sliding Distance.

#### Regression analysis

The importance of the parameters is evaluated by the use of ANOVA, which is then used to build the regression equation. The regression equation that illustrates the relationship between wear and other variables is shown in Eq. (1).

1 $${\text{Wear }}\left( {{\mathrm{Microns}}} \right)\, = \,{11}0.{148}{-}{4}.{15556}\; \times \;{\mathrm{Load}}\, + \,0.{1}0{8889}\; \times \;{\mathrm{Speed}} - 0.0{111111}\; \times \;{\text{Sliding Distance}} \ldots$$

As the value of sliding distance variable is insignificant its effect on wear can be neglected.

Therefore Eq. (1) can be rewritten as Eq. (2).

2 $${\text{Wear }}\left( {{\mathrm{Microns}}} \right)\, = \,{11}0.{148}{-}{4}.{15556}\; \times \;{\mathrm{Load}}\, + \,0.{1}0{8889}\; \times \;{\mathrm{Speed}} \ldots$$

#### Confirmation test

The confirmation test is the end step in the DOE method. Confirmation experiments were performed to gather data regarding the level of wear that the Al6061-Si_3_N_4_ composite has experienced. For the purpose of determining the optimal amounts of the variables for the S/N ratio, the main effect graph was applied^19^. The levels that are considered to be ideal for conducting confirmatory experiments are presented in Table 7.

**Table 7:** Optimum variables for wear confirmation test experiments.

Optimal parameters/factors	Load in N	Speed in rpm	Sliding distance in m
Wear	10	450	300

The results of the confirmatory examination experiments are displayed in Table 8. The confirmation tests outcomes showed that the error falls in an acceptable range and it is equal to 2.54%.

**Table 8:** Confirmatory experiment findings.

Response	OA experiment findings	Confirmation experimental findings	Error (%)
Wear in microns	115	118	2.54

**Fig. 8:**
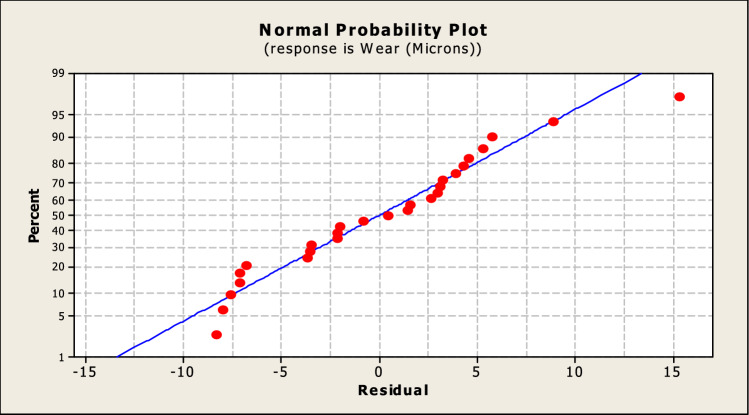
Normal probability plot.

A representation of the plot of the normal probability can be found in Fig. 8. The plot demonstrates that the errors caused by the experiment are not significant, as shown by the spots that are getting increasingly nearer to the line.

**Fig. 9:**
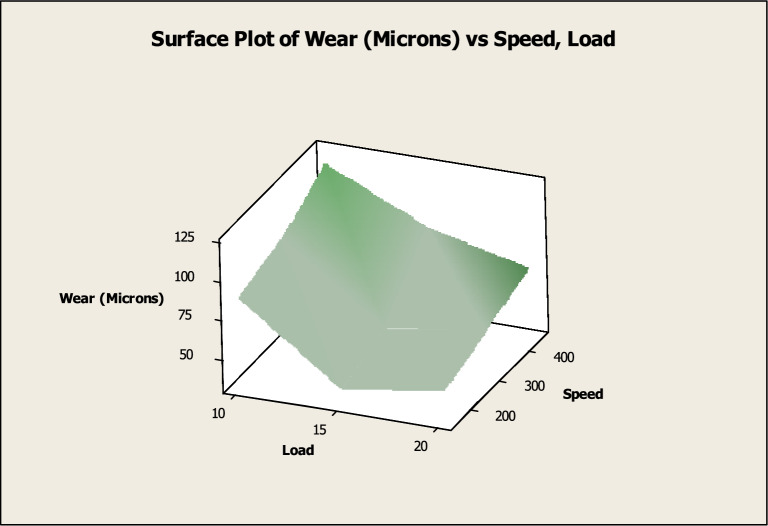
Surface diagram in relation to the load and speed.

**Fig. 10:**
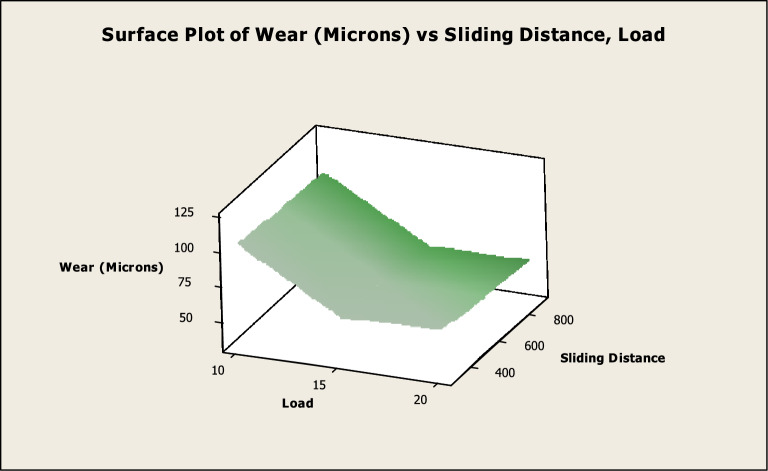
Surface diagram in relation to load and sliding distance.

Within the scope of this investigation, the wear performance was evaluated by means of 3D surface plot and are presented in Figs. 9 and 10.

The surface diagram of wear in connection to the applied load along with speed is depicted in Fig. 9. According to the graph that was just presented, the wear reduces up to 15N as the load rises, but then it begins to climb once more when the load reaches 20N. It has also been observed that the amount of wear rises in proportion to a rise in speed^22^. At 200 revolutions per minute, the wear is at its lowest, and at 400 revolutions per minute, it is at its highest. The surface diagram of wear in connection to the applied load along with sliding distance is indicated in Fig. 10. According to the graph that was just presented, the wear reduces up to 15N as the load rises, but then it begins to climb once more when the load reaches 20N. A further correlation exists between sliding distance length and the rate of wear that occurs^58^. An average sliding distance of 400 m results in the least amount of wear, while an average sliding distance of 800 m results in the most amount of wear.

## Conclusions

The following conclusions may be drawn from the present study on the fabrication, characterization and wear optimization of Al6061-6 wt. % Si_2_N_4_ composite:

- The microstructure of Al6061-6 wt. % Si_2_N_4_ composite revealed a less agglomerated homogeneous dispersion of Si_2_N_4_ particles.
- In Al6061 alloy, the adhesive wear mechanism was characterized by plastic deformation with deep grooves and delamination, while in Al6061-6 wt. % Si_2_N_4_ composite, adhesive to light abrasive wear with fine grooves and moderate micro ploughing was observed.
- The wear resistance of Al6061-6 wt.% Si_2_N_4_ composite was 21% higher than that of Al6061 alloy under optimal test conditions (load: 10 N, sliding speed: 450 rpm, and sliding distance: 300 m).
- According to statistical analysis using ANOVA, contact stress and subsurface deformation are directly impacted by the applied load, which is the primary factor influencing the wear.
- The Taguchi L27 optimization showed a percentage contribution of 63.2% for load, 30.4% for speed and 1.2% for sliding distance.
- The error in the confirmation experiments was within an acceptable range of 2.54%, and the regression model generated had a reasonable predictive capacity.

### Future work

- In order to broaden the impact and applicability of foreseeable research, future investigation may be considered in the following directions.
- Examination of the T6 and other heat-treated conditions in order to assess the synergistic effect of precipitation hardening with Si_3_N_4_ reinforcement on wear resistance and mechanical behavior.
- Long-term wearing tests under higher loading, longer sliding distance and higher temperature to study the progress of wear and stability of tribolayer.
- Research imperatives for hybrid reinforcements (Si_2_N_4_ combined with B_4_C, TiB₂ or SiC) that optimize wear resistance-versus-toughness optimization by considering the economic barrier.
- Combining Response Surface Methodology with replication tests to improve predictive capability, confirm ANOVA analyses and carry out multi-objective optimization.

## Data Availability

The datasets used and/or analyzed during the current study are available from the corresponding authors on reasonable request.

## References

[1] Beldar, P., Kadbhane, S. & Kavale, P. Microstructural and tribological investigation of B_4_C-reinforced Al6061 composites: Insights from SEM and wear analysis. *J. Eng. Appl. Sci.* **72**, 189. 10.1186/s44147-025-00769-8 (2025).

[2] Yadav, M., Kumaraswamidhas, L.A., Kumar, A. (2025). Study of Al-based composites reinforced with Al_2_O_3_ and WC: mechanical and surface properties, Journal of Adhesion Science and Technology, 1–26, 10.1080/01694243.2025.2555668

[3] Muhammed Muneer, S., Arun, B. S. & Vijay, R. Microstructure and mechanical properties of AlNbTaZr-Al_2_O_3_ refractory high entropy alloy reinforced Al6061 metal matrix composite. *Results in Surfaces and Interfaces* **20**(100622), 1–11. 10.1016/j.rsurfi.2025.100622 (2025).

[4] Lal, C., Tejyanb, S. & Singh, V. Fabrication and sliding wear characterization of eggshell particulate reinforced AA6061 alloy metal matrix composites. *Tribology in Industry* **46**(1), 29–38. 10.24874/ti.1483.05.23.08 (2024).

[5] Morampudi, P., Venkata Ramana, V. S. N., Somayajula, V. S. P. Microstructural and mechanical characterization of Al6061-ZrB_2_ nanocomposites fabricated by powder metallurgy. Journal of the Mechanical Behavior of Materials. 34, 20240033. 10.1515/jmbm-2024-0033 (2025).

[6] Sathiyaraj.S, Venkatesan Sendrayan, Kumaran Pachaiyappan (2025). Optimization of Wear Behaviour of Al6061 Metal Matrix Composites Using Taguchi Approach. Turkish Journal of Engineering. 9 (1), 56–63.

[7] Sreenivasa Iyengar, S.R., Sethuramu, D., Ravikumar, M. (2023). Study on hardness, fracture behavior and optimization of wear characteristics of Al6061/TiB_2_/CeO_2_ MMCs using Taguchi method, 64 178–193.

[8] Vijayakumar, R., Srikantamurthy, J. S., Patil, S. Optimization of wear process parameters of Al6061-Zircon composites using Taguchi method. Advances in Materials Science and Engineering. 1–10. 10.1155/2023/9507757 (2023).

[9] Tirfe, D., Debebe, A., Woldeyohannes, Regassa Hunde, B. Optimizing the dry sliding wear behavior of stir-casted Al6061/Nano-Al_2_O_3_/quartz hybrid metal matrix composite using Taguchi method. Advances in Tribology Volume, 1–14, 10.1155/2024/3589701 (2024).

[10] Ulmek, N. B. & Kumar, N. Study of CNC machining process parameters for Al 6061 reinforced with SiC and fly ash composites using Taguchi technique. *Evergreen Joint Journal of Novel Carbon Resource Sciences & Green Asia Strategy* **11**(04), 1–10 (2024).

[11] Mantha, S. R. V., Kumar, G. B. V. & Pramod, R. Investigations on microstructure, mechanical, and wear properties, with strengthening mechanisms of Al6061-CuO composites. *J. Manuf. Mater. Process.* **8**, 245. 10.3390/jmmp8060245 (2024).

[12] Sreenivasa Iyengar, S.R., Sethuramu, D., Ravikumar, M. (2023). Study on hardness, fracture behavior and optimization of wear characteristics of Al6061/TiB_2_/CeO_2_ MMCs using Taguchi method, 64, 178–193.

[13] Gowrishankar, T. P., Umesh, G. L., Vinod, B. R. & Ravikumar, M. Studies on mechanical, fractured surface, wear, and thermal characteristics of TiC reinforced structural grade Al6061 MMCs. *Fracture and Structural Integrity* **74**, 373–384 (2025).

[14] Beldar, P., Kadbhane, S. & Kavale, P. Microstructural and tribological investigation of B_4_C-reinforced Al6061 composites: Insights from SEM and wear analysis. *J. Eng. Appl. Sci.* **72**(189), 1–24. 10.1186/s44147-025-00769-8 (2025).

[15] Nagesh, D., Raghavendra, S. & Sivaram, N. M. Tribological characteristics of Al6061, boron, and graphite hybrid metal matrix composites. *Advances in Materials and Processing Technologies*10.1080/2374068X.2021.1946323 (2021).

[16] Muthu, P. An investigation on dry sliding wear behavior of Al6061/Titanium carbide/ Basalt hybrid metal matrix composites. *Incas Bulletin* **13**(4), 139–150 (2021).

[17] Gonfa, B. K., Sinha, D. & Vates, U. K. Investigation of mechanical and tribological behaviors of aluminum based hybrid metal matrix composite and multi-objective optimization. *Materials* **15**(5607), 1–18. 10.3390/ma15165607 (2022). 10.3390/ma15165607 PMC9413696 36013744

[18] Timish Bhave, Amol Borade, Diksha Bhoye (2020). Fabrication and tribological study of AA601 hybrid metal matrix composites reinforced with B_4_C nanoparticles, International Research Journal of Engineering and Technology (IRJET), Volume: 07 Issue: 06, 779–784.

[19] Sreenivasa Iyengar, S. R., Sethuramu, D. & Ravikumar, M. Study on hardness, fracture behavior and optimization of wear characteristics of Al6061/TiB_2_/CeO_2_ MMCs using Taguchi method. *Frattura ed Integrità Strutturale* **65**, 178–193. 10.3221/IGF-ESIS.65.12 (2023).

[20] Padmaraj, N. H., Keni, L. G. & Chethan, K. N. Sliding wear characteristics of boron carbide and novel squid quill ash reinforced aluminum 6061 hybrid composites. *J. Appl. Eng. Sci.*10.5937/jaes0-34487 (2022).

[21] Dakarapu, S. R. & Nallu, R. Process parameters optimization for producing AA6061/TiB_2_ composites by friction stir processing. *J. Mech. Eng.* **67**(1), 101–118. 10.1515/scjme-2017-0011 (2017).

[22] Singh, P. & Gupta, V. Dry sliding wear behaviour of al6061 hybrid metal matrix composites using response surface methodology. In: *IOP Conf.erence Series: Materials Science and Engineering* 1248, 012075 10.1088/1757-899X/1248/1/012075 (2022).

[23] Nagula, R. K., Dr, K. & Reddy, V. K. Tribological performance analysis of hybrid Al6061/Si_3_N_4_/SSP/RHA composite using Taguchi approach. *Int. J. Adv. Res. Eng. Technol. (IJARET)* **11**(9), 1272–1286. 10.34218/IJARET.11.9.2020.126 (2020).

[24] Manu Sama, N., Radhika, V. S. & Mohanraja, T. Investigation on the mechanical and wear behaviour of Al6082-BN-B_4_C-Corn. *Cob Ash Hybrid Comp. Tribol. Ind.* **22**(2), 294–309. 10.24874/ti.1165.08.21.11 (2021).

[25] Moustafa, E. B. Multi-functional AA6061 alloy hybrid composites via friction stir processing: Tailoring properties with TiNi and ceramic nanoparticles. *Compos. Interfaces* **32**(6), 931–952. 10.1080/09276440.2024.2446846 (2024).

[26] Singh, A. et al. Tribological performance and surface morphological analysis of in-situ synthesized BN-Si_3_N_4_ reinforced SiC-Al_2_O_3_ ceramic matrix composites. *Tribol. Int.* **209**, 110703. 10.1016/j.triboint.2025.110703 (2025).

[27] Shen, C. et al. Physical metallurgy-guided machine learning and artificial intelligent design of ultrahigh-strength stainless steel. *Acta Mater.* **179**, 201–214. 10.1016/j.actamat.2019.08.033 (2019).

[28] Awate, P. P. & Barve, S. B. Microstructural observation and mechanical properties behavior of Al_2_O_3_/Al6061 nanocomposite fabricated by stir casting process. *Eng. Res. Express* **4**(1), 015023. 10.1088/2631-8695/ac54ed (2022).

[29] Kumar, R., Keshavamurthy, R. & Perugu, C. S. Influence of hot rolling on friction and wear behaviour of Al6061-ZrB_2_ in-situ metal matrix composites. *J. Manuf. Process.* **69**, 473–490. 10.1016/j.jmapro.2021.07.058 (2021).

[30] Tejyan, S., Ror, C. K. & Kumar, N. Mechanical properties of SiC and neem leaf powder reinforced Al-6063 hybrid metal matrix composites. *Mater. Today Proceed.* **60**, 884–888. 10.1016/j.matpr.2021.09.521 (2022).

[31] Kumar, V. et al. Physico-tribomechanical performance of Zn-Al/ZrB_2_ in-situ composite for sustainable automotive applications. *Tribol. Int.* **206**, 110591. 10.1016/j.triboint.2025.110591 (2025).

[32] Zeng, L. et al. Study on dynamic wear evolution of modified gear rack considering the real-time variation of contact characteristics. *Wear* **571**, 205845. 10.1016/j.wear.2025.205845 (2025).

[33] Wang, S. et al. Multifunctional tribovoltaic coating for self-powered in situ sensing with exceptional tribological robustness and charge transport. *Adv. Func. Mater.*10.1002/adfm.202514190 (2025).

[34] Sivamaran, V., Balasubramanian, V. & Gopalakrishnan, M. Mechanical and tribological properties of Self-Lubricating Al6061 hybrid nano metal matrix composites reinforced by nSiC and MWCNTs. *Surf. Interfaces.* **21**, 100781 (2020).

[35] Boppana, S. B., Dayanand, S. & Kumar, M. A. Synthesis and characterization of nano graphene and ZrO_2_ reinforced Al6061 metal matrix composites. *J Mater Res Technol.* **9**(4), 7354–7362 (2020).

[36] Choi, S. K., Seo, B. & Kang, J. W. Microstructure and wear properties of aluminum metal matrix composite (Al6061-B_4_C) fabricated by stir casting process. *Arch. Metall. Mater.* **70**(2), 601–607. 10.24425/amm.2025.153461 (2025).

[37] Abebe Emiru, A., Sinha, D. K. & Kumar, A. Fabrication and characterization of hybrid aluminum (Al6061) metal matrix composite reinforced with SiC, B_4_C and MoS_2_ via stir casting. *Int. J. Met.*10.1007/s40962-022-00800-1 (2022).

[38] Kumar, V., Singh, A., Ankit, & Gautam, G. A comprehensive review of processing techniques, reinforcement effects, and performance characteristics in copper-based metal matrix composites. *Interactions*10.1007/s10751-024-02200-9 (2024).

[39] Qiao, Q. et al. In-situ monitoring of additive friction stir deposition of AA6061: Effect of rotation speed on the microstructure and mechanical properties. *Mater. Sci. Eng. A* **902**, 146620. 10.1016/j.msea.2024.146620 (2024).

[40] Yunus, M. & Alfattani, R. Assessment of mechanical and tribological behavior of AA6061 reinforced with B_4_C and Gr hybrid metal matrix composites. *Coatings* **13**, 1653 (2023).

[41] Kumar, V., Kushwaha, S., Ankit, A. & Sharma, G. Microstructural and mechanical analysis of heat-treated Zn-Al alloy and Zn-Al/ZrB_2_ composites. *Mater. Lett.* **366**, 136573. 10.1016/j.matlet.2024.136573 (2024).

[42] Peng, J. et al. Effect of the activator B(OCH_3_)_3_ on the microstructure and mechanical properties of Cu−Mn−Al alloy coating via CMT cladding. *Crystals* **15**(10), 881. 10.3390/cryst15100881 (2025).

[43] Ashok Kumar, B., Muthu Krishnan, M. & Felix Sahayaraj, A. Characterization of the aluminum matrix composite reinforced with silicon nitride (AA6061/Si_3_N_4_) synthesized by the stir casting route. *Adv. Mater. Sci. Eng.*10.1155/2022/8761865 (2022).

[44] Arya, R. K., Kumar, R. & Telang, A. Influence of microstructure on tribological behaviors of Al6061 metal matrix composite reinforced with silicon nitride (Si_3_N_4_) and silicon carbide (SiC) micro particles. *SILICON* **15**, 3987–4001. 10.1007/s12633-023-02309-6 (2023).

[45] Kumaraswamy, J., Bharath, L., Anil, K. C., Geetha, T. M. & Nagesh, R. Results in mechanical properties and wear behaviour of AA6061-Si_3_N_4_ composites. *Results Surf. Interfaces* **18**, 100376. 10.1016/j.rsurfi.2024.100376 (2025).

[46] Kumar, V., Mishra, A., Mohan, S. & Mohan, A. Fabrication of stircast ZA/ZrB2 reinforced in-situ composites. *Mater. Res. Expr.* **6**, 126555. 10.1088/2053-1591/ab53f2 (2019).

[47] Ankit, V. K., Yadav, A. K., Gautam, G., Singh, K. K. & Mohan, S. Prediction of tribological performance of Cu-Gr-TiC composites based on response surface methodology and worn surface analysis. *Phys. Scr.* **98**, 115971. 10.1088/1402-4896/acff8d (2023).

[48] Paul, P., Ghosh, S. K. & Nath, R. K. Parametric study and characterization of SiC–TiC–Al composite coatings fabricated on Al6061 alloy by TIG cladding process. *SILICON* **17**, 3317–3332. 10.1007/s12633-025-03418-0 (2025).

[49] Yuvaraj, N., Koli, Y. & Vedabouriswaran, G. Mechanical and tribological properties of AA6061/SiC/Aloe Vera powder hybrid Al composites fabricated by stir casting. *SILICON* **15**, 2451–2465. 10.1007/s12633-022-02168-7 (2023).

[50] Rawal, S. & Sidpara, A. M. Tool wear evaluation in micro-milling of Al6061/GNPs nanocomposite. *Wear*10.1016/j.wear.2025.206269 (2025).

[51] Koti, V. et al. Fabrication and wear characteristics of al6066-boron carbide particulate composites. *J. Inst. Eng. India Ser. D*10.1007/s40033-025-00887-w (2025).

[52] Jagadeesha, T. et al. Investigation of tribological behavior of fused deposition modelling processed parts of polyethylene terephthalate glycol polymer material. *J. Inst. Eng. India Ser. D.*10.1007/s40033-025-00885-y (2025).

[53] Junaidi, M. Influence of zirconium carbide particles on the mechanical characteristics of heat treated Al7475 alloy. *Sci. Rep.* **15**, 15011. 10.1038/s41598-025-99221-3 (2025). 40301451 10.1038/s41598-025-99221-3 PMC12041601

[54] Hariharana, S. R., Sakthia, S., Mahendranb, S. & Jayasuthakar, S. T. Influence of MgO and SiC on mechanical and wear characteristics of AA6061 hybrid matrix composites synthesized by stir casting. *J. Appl. Res. Technol.* **21**, 162–168 (2023).

[55] Vijaya Kumar, R., Hemanth Raju, T. & Udayashankar, S. Experimental investigation of Al6063 alloy with zirconium silicate composite. *J. Inst. Eng. India Ser. D*10.1007/s40033-024-00643-6 (2024).

[56] Guo, J., Han, Y. & Xiao, Ke. Evaluation of linear and nonlinear methods for predicting the dynamic behavior of plain and textured water-lubricated bearings. *Phys. Fluids* **37**, 083621. 10.1063/5.0280770 (2025).

[57] Kumar, V., Gautam, G., Ankit, A. M. & Mohan, S. Correlating surface topography of relaxed layer of ZA/ZrB_2_ in situ composites to wear and friction. *Surf. Topogr. Metrol. Prop.* **11**, 025006. 10.1088/2051-672X/acc881 (2023).

[58] Ranjitha, P., Hemanth Raju, T. & Udayashankar, S. Study of wear behavior of silicon carbide and boron carbide reinforced alumiium alloy (Al6061) matrix composites. *J. Inst. Eng. India Ser. D*10.1007/s40033-023-00625-0 (2023).

